# Dysregulation of Leukocyte Trafficking in Type 2 Diabetes: Mechanisms and Potential Therapeutic Avenues

**DOI:** 10.3389/fcell.2021.624184

**Published:** 2021-02-22

**Authors:** Laleh Pezhman, Abd Tahrani, Myriam Chimen

**Affiliations:** ^1^Institute of Inflammation and Ageing, College of Medical and Dental Sciences, University of Birmingham, Birmingham, United Kingdom; ^2^Institute of Metabolism and Systems Research, University of Birmingham, Birmingham, United Kingdom; ^3^Centre for Endocrinology, Diabetes and Metabolism, Birmingham Health Partners, Birmingham, United Kingdom; ^4^University Hospitals Birmingham NHS Foundation Trust, Birmingham, United Kingdom

**Keywords:** therapies, inflammation, type 2 diabetes, obesity, trafficking, leukocyte

## Abstract

Type 2 Diabetes Mellitus (T2DM) is a chronic inflammatory disorder that is characterized by chronic hyperglycemia and impaired insulin signaling which in addition to be caused by common metabolic dysregulations, have also been associated to changes in various immune cell number, function and activation phenotype. Obesity plays a central role in the development of T2DM. The inflammation originating from obese adipose tissue develops systemically and contributes to insulin resistance, beta cell dysfunction and hyperglycemia. Hyperglycemia can also contribute to chronic, low-grade inflammation resulting in compromised immune function. In this review, we explore how the trafficking of innate and adaptive immune cells under inflammatory condition is dysregulated in T2DM. We particularly highlight the obesity-related accumulation of leukocytes in the adipose tissue leading to insulin resistance and beta-cell dysfunction and resulting in hyperglycemia and consequent changes of adhesion and migratory behavior of leukocytes in different vascular beds. Thus, here we discuss how potential therapeutic targeting of leukocyte trafficking could be an efficient way to control inflammation as well as diabetes and its vascular complications.

## Introduction

**FIGURE 1:**
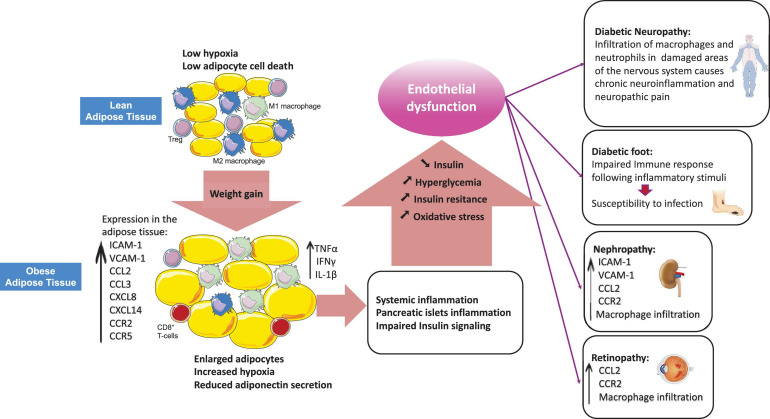
Obesity-related dysregulation of leukocyte trafficking in T2DM. Adipose tissue from lean individuals is characterized by increased numbers of anti-inflammatory M2 macrophages and higher presence of regulatory T-cells (Tregs). Obesity induces adipose tissue enrichment of cytotoxic CD8^+^ T-cells and pro-inflammatory M1 macrophages with a shift from an anti-inflammatory to a pro-inflammatory state. In obesity, the imbalance among leukocytes results in production of inflammatory cytokines (TNFα, IFNγ, IL-1β) and chemokines which promote systemic inflammation and peripheral insulin resistance as well as beta-cell dysfunction. Hyperglycemia occurs as a result of beta-cells failure to compensate for insulin resistance. Hyperglycemia exacerbates systemic inflammation by inducing pro-inflammatory cytokines production and modulating leukocyte trafficking through alteration of the expression of adhesion molecules, chemokines and chemokine receptors in different vascular beds, which ultimately results in vascular complications.

Inflammation is a protective response against harmful stimuli such as injuries, infections and toxins, which requires the trafficking of leukocytes from the blood stream to the site of inflammation within damaged tissues. This process is critical for elimination of harmful stimuli and for tissue repair and is tightly regulated by a variety of mediators such as cell adhesion molecules, cytokines and chemokines (Wright and Cooper, 2014). Leukocyte behavior and their recruitment to tissues is modified under inflammatory conditions such as in Type 2 diabetes (T2DM) (Wu et al., 2011). T2DM is a chronic inflammatory disorder characterized by hyperglycemia and impaired insulin signaling and production (Calle and Fernandez, 2012). Components of the immune system are altered in T2DM, with the most apparent changes occurring in the adipose tissue, the pancreatic islets, the vasculature and in circulating leukocytes (Donath and Shoelson, 2011; Mraz and Haluzik, 2014; Eguchi and Nagai, 2017). Indeed, cellular stresses, such as oxidative stress and lipotoxicity cause insulin resistance and pancreatic islets dysfunction, can induce an inflammatory response and also be exacerbated by local inflammation. Patients with T2D have elevated levels of inflammatory cytokines (such as IL-1β and IL-6) and chemokines (such as CCL2, CXCL8) (Donath and Shoelson, 2011). This rise in inflammatory mediators completely modifies leukocyte behavior and recruitment into tissues, which in turn contributes to maintenance of insulin resistance, loss of insulin secretion and accelerates development of micro and macro-vascular complications (Wu et al., 2011). Clinical studies show that targeting inflammation using small molecules or biological agents to turn off pro-inflammatory cytokines, deplete immune cells or block regulatory surface receptors, results in improved blood glucose levels and insulin sensitivity (Larsen et al., 2007; Donath and Shoelson, 2011). Obesity, particularly visceral adiposity, is a major risk factor for T2DM (Donath and Shoelson, 2011) and the adipose tissue of patients with T2DM and/or obese patients appears to be a major site of inflammation (Lontchi-Yimagou et al., 2013). Both innate and adaptive immune cells present in the adipose tissue have critical roles in the regulation of metabolic homeostasis. Dysregulated trafficking of leukocytes is observed in the adipose tissue and is associated with a shift in cell populations from an anti-inflammatory to a pro-inflammatory profile in obesity (Esser et al., 2014). This switch to a production of pro-inflammatory cytokines leads to the development of systemic low-grade inflammation, which results in impaired insulin signaling, beta-cell dysfunction and subsequent insulin resistance (Esser et al., 2014). Hyperglycemia rises as the pancreas fails to compensate insulin resistance (Donath and Shoelson, 2011). Hyperglycemia then leads to oxidative stress and induces systemic inflammation by stimulating the production of inflammatory cytokines (Noshita et al., 2002; Dimayuga et al., 2007) and chemokines, resulting in endothelial dysfunction (Nguyen et al., 2012). This review will summarize how T2DM-related changes in the expression of adhesion molecules and chemokine receptors/ligands drive endothelial dysfunction and dysregulates leukocyte trafficking under inflammatory conditions which ultimately leads to diabetic complications (Figure 1).

## The Molecular Processes of Leukocyte Recruitment in Inflammation

Recruitment of leukocytes is triggered by the local release of inflammatory mediators such as histamine, tumor necrosis factor-alpha (TNFα) and interferon-gamma (IFNγ) in inflamed tissue (Liu et al., 2004; Zhang et al., 2011; Muller, 2013). These mediators induce the expression of specific adhesion receptors such as selectins, adhesion molecules and chemokines, which are expressed or presented at the surface of endothelial cells (ECs) and bind to integrins or chemokine receptors located at the surface of leukocytes (Zhang et al., 2011). Selectins interaction with their ligands results in leukocyte tethering and rolling from rapidly flowing blood (Kansas, 1996; Harjunpää et al., 2019). Selectins are transmembrane calcium-dependent lectins that consist of three functionally different adhesion molecules expressed by ECs (E-selectin), platelets and ECs (P-selectin) and leukocytes (L-selectin) (McEver, 2015). L-selectin and P-selectin mediate fast rolling of leukocyte through sequential interactions with their ligands. However, slow rolling events are mediated by E-selectin on ECs binding to E-selectin ligand-1 (ESL-1) or P-selectin glycoprotein ligand 1 (PSGL-1) on leukocytes (Yago et al., 2010). Leukocyte arrest and migration on the endothelium depends on chemokines (Luu et al., 2000). The shift from fast rolling to slow rolling allows G-protein-coupled receptors (GPCRs) on leukocytes to bind to the chemokines expressed on the surface of ECs, which induces leukocyte integrin activation and enables leukocyte firm adhesion to the vessel wall (Giagulli et al., 2004; Rutledge and Muller, 2020). Integrins are a family of transmembrane receptors expressed by leukocytes. Both α_4_β_1_ integrin or very late antigen-4 (VLA-4) and α_M_β_2_ integrin or lymphocyte function-associated antigen-1 (LFA-1) have been found to play a role in leukocyte arrest (Uotila et al., 2014). α_M_β_2_ is expressed on leukocytes as a resting low affinity state. Conformational changes are required for integrins to switch from a low affinity state to an active high affinity state enabling them to bind to their ligands expressed on the endothelium (Kim et al., 2003; Ley et al., 2007; Fan and Ley, 2015). The activation of integrins is mediated by chemokines and results in integrin extension and changes in the cytoskeleton of leukocytes, enabling tight adhesion on the vessel wall (Rutledge and Muller, 2020). LFA-1 and VLA-4 on leukocytes, bind to their respective ligand ICAM-1 and VCAM-1 on ECs, mediate leukocyte adhesion (Hyduk et al., 2007; Kuwano et al., 2010) and induce intracellular signals to allow leukocyte arrest (Giagulli et al., 2006; Kummer and Ebnet, 2018). Leukocytes then extend lamellipodia and prepare for transendothelial migration (Rutledge and Muller, 2020). Additional downstream signals such as prostaglandin-D2 (PGD2) signals are required for shape change and dynamic transmigration of activated leukocytes (Ahmed et al., 2011). Macrophage integrin-1 (Mac-1) or CD11b/CD18, also known as α_M_β_2_, interaction with ICAM-1 expressed by the inflamed endothelium is required for the transition of leukocytes from tight adhesion to crawling along the vessel wall (leukocyte locomotion) to then find a suitable site for transendothelial migration (Schenkel et al., 2004). Leukocytes either transmigrate (diapedesis) through the endothelial junctions (paracellular route), or through the body of the ECs (transcellular route) (Ley et al., 2007). In the paracellular route, the distribution of junctional molecules is modified in inflamed ECs in a way that facilitates transendothelial cell migration (Shaw et al., 2001). Junctional molecules such as platelet/endothelial-cell adhesion molecule 1 (PECAM-1) and junctional adhesion molecule A (JAM-A) may support leukocyte migration through mobilizing to the luminal surface where they can bind to their ligands expressed on leukocytes and guide them to the junctions (Muller, 2003). JAM-A interaction with α_L_β_2_ allows strong adhesion of activated leukocyte to the endothelium (Kummer and Ebnet, 2018). PECAM-1 is expressed at EC junctions and leukocytes (Newman et al., 1990). When leukocytes reach the site of transmigration, the homophilic interactions between PECAM-1 on leukocytes with PECAM-1 on the endothelium causes a transient increase in cytosolic calcium in ECs which guide the leukocytes to the junctions (Ley, 2007; Muller, 2011). Like PECAM-1, CD99 (Schenkel et al., 2002) and CD99L2, expressed at EC junctions and on leukocytes, rely on a homophilic interactions to promote leukocyte extravasation (Schenkel et al., 2002; Seelige et al., 2013; Rutledge et al., 2015). In ECs, the major functional component of the adherent junction is VE-cadherin. During transendothelial migration, there is a transient gap in VE-cadherin at the site of the transmigrating leukocytes, which facilitates transmigration (Dejana and Giampietro, 2012). This gap has been shown to be triggered by constitutive shedding of VE-cadherin by ADAM-10, which is promoted by Tspan5 and Tspan17 and therefore allows T-cell diapedesis (Reyat et al., 2017).

Transcellular leukocyte migration only occurs for a minority of emigrating cells and mostly takes place in thin parts of the endothelium (Carman and Springer, 2004). In this route, leukocyte migration occurs through membrane-associated channels, which act as a gateway for leukocytes through the body of ECs. In fact, the preferred route of leukocyte transmigration depends on the leukocyte type and also the type of vascular bed (Dvorak and Feng, 2001). The endothelium is heterogeneous in different vascular beds, depending on the size and the organ considered (Aird, 2007). ECs in the arterioles of the vascular system have lower permeability to leukocytes, whereas those in the venules are thinner and permissive allowing leukocytes to easily transmigrate particularly in postcapillary venules (Dejana et al., 2009). In addition, the endothelial barrier can be even more permissive such as in lymphoid organs (Miyasaka and Tanaka, 2004), or very tight such as in the central nervous system (Wolburg and Lippoldt, 2002).

To reach inflamed tissues, leukocytes need to migrate through the basement membrane. The basement membrane is made up of laminin and collagen type IV (Mutgan et al., 2020). In murine models, leukocytes cross the basement membrane at areas with low expression of laminin (Song et al., 2017). PECAM-1, CD99, and CD99L2 are required for homophilic interactions between leukocyte and ECs to initiate the migration through the basement membrane (Thompson et al., 2001; Bixel et al., 2010; Sullivan et al., 2016). Leukocytes express several membrane-type matrix metalloproteinases (MMP) including the secreted- and membrane-anchored MT-MMPs, which develop an appropriate proteolytic reaction (Marco et al., 2013) and enable cells to cross this structural barrier. However, the exact mechanisms still remain to be fully characterized. Finally, following a successful diapedesis, leukocytes utilize a chemokine gradient to migrate toward the site of infection upon their entry into inflamed tissues (Ley et al., 2007).

## Expression of Adhesion Molecules in T2DM

Cell adhesion molecules (CAMs) are glycoproteins expressed on the surface of various cell types such as ECs and leukocytes (Galkina and Ley, 2007). Upon inflammation, vascular ECs express or up-regulate the expression of CAMs that increase the attachment of leukocytes to the endothelium (Cook-Mills et al., 2011). Selectins, ICAM-1 and VCAM-1 are the major CAMs responsible for leukocyte adhesion (Huo and Ley, 2001). Soluble form of CAMs (sCAMs) in the circulation indirectly reflect the rate of endothelial expression and activation of CAMs since they are shed from the surface of ECs and lymphocytes after being activated (Abe et al., 1998). Elevated expression and activity of CAMs are therefore indicative of inflammation and endothelial dysfunction (Cook-Mills et al., 2011).

### Soluble CAMs and Selectins

The baseline levels of circulating E-selectin, ICAM-1, and VCAM-1 were found higher in patients with T2DM compared to healthy controls (Meigs et al., 2004). A study on 150 Japanese patients with T2DM showed that higher serum concentrations of sVCAM-1 and sE-selectin were positively correlated with fasting plasma glucose levels, and negatively correlated with insulin sensitivity (Matsumoto et al., 2002). Higher levels of circulating CAMs in T2DM is linked to an increased production of advanced glycosylation end products (AGEs) and oxidative stress occurring under hyperglycemic conditions (Hadi and Al Suwaidi, 2007). The interaction of AGEs with the vessel wall components increases the generation of reactive oxygen species, which results in an increase in the surface expression of CAMs on activated ECs (Wen et al., 2002; Basta et al., 2004; Farhangkhoee et al., 2006).

Hyperinsulinemia can also directly affect the surface expression of adhesion molecules on ECs from healthy volunteers and from patients with non-insulin-dependent diabetes *in vitro* (Okouchi et al., 2002). Culture of ECs in insulin-rich medium (over 50 microUnit/ml) for 24 h increased the surface expression of PECAM-1 but not of ICAM-1, P-selectin or E-selectin (Okouchi et al., 2002). This was clearly associated with an increase in neutrophil adhesion therefore suggesting that high insulin conditions promote vascular injury and therefore T2DM-associated macro- and micro-vascular complications.

However, a recent study on 58 patients with T2DM on insulin therapy and displaying microvascular complications found lower levels of serum sICAM-1 in these patients irrespective of the type of diabetic complication when compared to age-matched healthy controls (Hocaoglu-Emre et al., 2017). Since the participants with T2DM were also receiving angiotensin-converting enzyme (ACE)-inhibitor agents, decreased levels of sICAM-1 might have resulted from the combination of insulin and ACE-inhibitor therapies. Indeed, both insulin and ACE inhibitors can individually inhibit CAMs surface expression on ECs as well as their circulating levels (Drexler et al., 1995; Aljada et al., 2000). However much remain to be clarified in this area as the effect of both insulin and ACE inhibitors seems to vary in different patient cohorts. Some studies have hypothesized that these discrepancies between cohorts are linked to the degree of complications and the bigger impact seems to occur in late-stage diabetic complications when other treatments such as ACE-inhibitors are used. The conflicting evidence about the levels of sCAMs in the circulation suggests that different CAMs may play different roles in the different stages of vascular complications in T2DM (Hocaoglu-Emre et al., 2017).

### Surface Expression of CAMs on the Endothelium

Variations in the expression of adhesion molecules on the vasculature play a role in microangiopathy in patients with T2DM by enhancing leukocyte adhesion in the vasculature and causing capillary obstruction (McLeod et al., 1995). This has been observed in different vascular beds and is linked to a majority of complications such as diabetic nephropathy (Gu et al., 2013) and retinopathy (McLeod et al., 1995). A study on 40 patients with T2DM showed a significant increase in the expression of ICAM-1 and VCAM-1 on vascular ECs from the conjunctiva in comparison with conjunctiva from healthy controls (Khalfaoui et al., 2008). Confocal microscopy imaging of retina vessels in streptozotocin (STZ)-induced hyperglycemic mice reported increased VCAM-1 protein levels after 8 weeks, and this was synchronized with the expression of the inflammatory cytokines TNFα, IL-6 and interleukin 1 beta (IL-1β) in the retina (Gustavsson et al., 2010). However, not many studies have performed in depth study of leukocyte recruitment in patients and in mice models to show that defects in leukocyte recruitment could mean leukocyte trafficking is impaired despite upregulation of endothelial CAMs.

Vascular adhesion protein-1 (VAP-1) is an enzyme and an adhesion molecule mainly expressed by ECs, smooth muscle, and the adipose tissue (Kuo et al., 2019). Endothelial VAP-1 supports leukocyte rolling, firm adhesion, and transmigration (Salmi and Jalkanen, 2019). The catalytic activity of VAP-1 can be damaging and cytotoxic to ECs via the generation of AGEs, which are involved in the pathogenesis of diabetic complications such as retinopathy, nephropathy, neuropathy, and atherosclerosis (Stolen et al., 2004).

Vascular adhesion protein-1 expressed on the vessels in the pancreatic islet of Non-Obese Diabetic (NOD) mice (model of T1DM) was associated with high degree of lymphocyte infiltration into the islets (Bono et al., 1999). Invalidation of the gene encoding VAP-1, using a null mutation in the amine oxidase copper-containing-3 (AOC3), decreased infiltration of T-cells, macrophages and NK cells in both epididymal and inguinal white adipose tissue of AOC3-KO mice compared to age-matched wild-type controls. This reduction in leukocyte infiltration was associated to a reduced capacity of leukocyte extravasation normally enabled via VAP-1 (Jargaud et al., 2020). Serum VAP-1 levels are higher in patients with T2DM and in patients with chronic kidney disease, which makes serum VAP-1, a good predictor of end-stage renal disease in diabetic patients and a useful biomarker to improve risk stratification of patients with T2DM (Li et al., 2016). Many *in vivo* studies further validate the role of VAP-1 as an anti-inflammatory target (Xu et al., 2006; Foot et al., 2013; Carpéné et al., 2019). Indeed, administration of a long-lasting VAP-1 and 2 inhibitor, PXS-4681A, in a mouse model of LPS-induced lung inflammation attenuated neutrophil migration into the lungs (Foot et al., 2013). PXS-4681A is a promising drug candidate as it ensures complete and long-lasting inhibition of the enzymes after a single low dose *in vivo* and could also be tested in other models of chronic inflammation.

### Surface Expression of CAMs on Leukocytes

Leukocyte adhesion to the endothelium is mediated by surface integrins expressed on leukocytes such as macrophage integrin-1 (Mac-1) or CD11b/CD18, also known as α_M_β_2_ (Carlos and Harlan, 1994). In a study comparing patients with T2DM to age-matched healthy controls, basal expression of CD11b on monocytes and neutrophils did not differ between the groups (Sampson et al., 2002). However, higher expression of CD11b was observed on monocytes after a glucose load in both T2DM and control groups. This suggests, an association between hyperglycemia and monocyte-endothelial interactions through increased expression of monocytic CD11b (Sampson et al., 2002). Higher expression of CD11b in both diabetic and control groups after a glucose load could be due to the translocation of stored CD11b to the cell surface in response to any acute or sudden change in glucose levels regardless of basal plasma glucose (Sampson et al., 2002). However, another group later found higher monocyte expression of CD11b at baseline in patients with obesity, which may suggest higher degree of leukocyte activation with increased adhesive properties in this group compared to lean participants (Van Oostrom et al., 2004). This was not limited to monocytes as later de Vries et al. (2015) also found up-regulation of CD66b (glycosylated antigen implicated in adhesion to E-selectin) on neutrophils from patients with T2DM. Change in CD66 can be implicated in aberrant neutrophil recruitment to the vasculature but also indicates neutrophil activation and degranulation (Weber, 2003), which confirms how hyperglycemia can lead to exacerbated inflammatory responses.

Dipeptidyl peptidase-4 (DPP-4) or CD26 is an amino-peptidase expressed in numerous tissues including the vasculature and in immune cells (Mentlein, 1999; Lambeir et al., 2003). DPP-4 cleaves dipeptides from the N-terminus of many chemokines and cytokines, usually after a penultimate proline or an alanine (Broxmeyer et al., 2016). DPP-4 controls glucose homeostasis through regulating bioactivity of the incretin hormone and glucagon-like peptide 1 (Augustyns et al., 2010). Involvement of DPP-4 in metabolic control raises the possibility that it may play a role in metabolic diseases such as diabetes and obesity (Mulvihill and Drucker, 2014; Omar and Ahrén, 2014). Given its various roles and its altered expression and activity, DPP-4 has been implicated in several pathological processes, including inflammation, viral entry and immune-mediated diseases (Lambeir et al., 2003; Yu et al., 2010).

Plasma levels of DPP-4 and circulating DPP-4 activity both increase with obesity and this correlates with insulin resistance (Mulvihill and Drucker, 2014; Ahmed et al., 2017). Numerous studies have detailed the effects of DPP-4 inhibitors on insulin and/or glucagon secretion, but little evidence indicates that DPP-4 inhibitors directly improve chronic inflammation. The effects of DPP-4 inhibition was investigated in diet-induced adipose tissue inflammation using (Gck^+/2^) diabetic mice, an animal model of non-obese T2DM (Shirakawa et al., 2011). DPP-4 inhibition in this model led to a significant reduction in adipose tissue infiltration of CD8^+^ T-cells and M1 macrophages which was associated to decreased mRNA expression levels of TNF-α and MCP-1 in the adipose tissue (Shirakawa et al., 2011). Because DPP-4 mediates the cleavage of many chemokines and adipokines, inhibition of DPP-4 may cause off-target side effects and further research is needed to clarify this (Shirakawa et al., 2011).

In addition, Zhuge et al. (2016) found that the expression of DPP-4 was mainly detected and up-regulated on F4/80^+^ macrophages in the white adipose tissue of HFD-induced obese mice). Oral administration of the DPP-4 inhibitor, linagliptin, caused an anti-inflammatory macrophage polarization with a dynamic M2 shift of macrophages within the adipose tissue of HFD-induced obese mice compared to non-treated obese mice, and this contributed to the attenuation of whole-body insulin resistance (Zhuge et al., 2016). In this study, the anti-inflammatory effects of DPP-4 inhibition were due to a decreased in ROS generation and an attenuation of oxidative stress in the white adipose tissue (Zhuge et al., 2016). Loss of macrophage inflammatory protein-1α (MIP-1α), a chemokine and potential DPP-4 substrate, abrogated the M2 macrophage-polarizing and insulin-sensitizing effects of linagliptin in MIP-1α^–/–^ mice on HFD. This suggests that MIP-1α may be a substrate for DPP-4 and contributes to the regulation of macrophage polarization in obesity models (Zhuge et al., 2016).

Altogether the *in vivo* studies highlight a potential role of DPP-4 in the modulation of leukocyte migration by affecting chemokines. However, this has not been extensively investigated in the studies presented here as not all studies seem to have linked this to migration despite the important role of DPP-4 at inhibiting chemokines. The approved DPP4 inhibitors being used in clinic, such as sitagliptin and vildagliptin are based on the ability of DPP4 to lower glucose rather than its regulatory effects on inflammation (Deacon, 2018).

Similar to studies investigating endothelial expression of adhesion molecules in T2DM, data showing changes in adhesion molecules/integrins on leukocytes have not performed analysis of leukocyte recruitment in targeted tissues, instead this has been extrapolated from knowledge on adhesion molecule profiles and function in “normal” inflammatory responses, in other models and/or diseases.

## Expression of Chemokines and Chemokine Receptors in T2DM

Chemokines and their receptors play a central role in leukocyte trafficking and are involved in the pathophysiology of T2DM (Buraczynska et al., 2012). Production and release of cytokines and chemokines including CC-chemokine ligand 2 (CCL2), CCL3 and CXC-chemokine ligand 8 (CXCL8) is increased in the adipose tissue and pancreatic islets of patients with T2DM (Donath and Shoelson, 2011; Xu et al., 2015). This is associated with an increased recruitment of macrophages in these tissues, and thus contributes to tissue inflammation (Donath and Shoelson, 2011; Xu et al., 2015). CCL2 (also known as MCP-1) is known to regulate monocyte recruitment by directing monocytes migration from the bone marrow to inflamed tissues (Crane et al., 2009; Boels et al., 2017). The CCL2-CCR2 axis has a key role in diabetes-related complications such as retinopathy, nephropathy and neuropathy (White et al., 2009; Zhu et al., 2014; Moreno et al., 2018; Monickaraj et al., 2020). Indeed, contribution of CCL2-CCR2 axis to leukocytes recruitment into the injured nerve was shown using a preclinical model of peripheral neuropathic pain induced by chronic nerve constriction (CCI) in Sprague–Dawley rats (Van Steenwinckel et al., 2015). This study shows that CCl-induced mechanical hypersensitivity in rats upregulated the expression of CCL2 and increased local macrophage infiltration in the sciatic nerve compared to the sham group (Van Steenwinckel et al., 2015). In contrast, in the same study, CCL2-deficient mice presented attenuated CCI-induced mechanical hypersensitivity as well as decreased number of macrophages infiltrating the injured sciatic nerve. Altogether, these findings suggest that CCL2 could play an important role in mediating neuropathic pain by increasing leukocyte infiltration in nerves but also by mediating neuro-immune interactions during inflammation-induced pain (reviewed in White et al., 2009).

In addition, upregulation of the CCL2 gene was also demonstrated in retinas of STZ-induced diabetic rats, and this was coincident with the trafficking and infiltration of numerous monocytes into the retina (Rangasamy et al., 2014). Furthermore, using CCL2 knockout (*Ccl2^–/–^*) mice, the same authors found significantly decreased monocyte/macrophage trafficking into diabetic *Ccl2^–/–^* retinas, indicating that this chemokine may be essential for the alteration of the blood-retinal barrier (Rangasamy et al., 2014). The role of CCL2-CCR2 axis is also widely investigated in diabetic renal injuries. Indeed, accumulation of macrophages in the kidney was reduced in *Ccl2 ^–^*^/^*^–^ db*/*db* double knock-out diabetic mice compared to *Ccl2* ^+/+^ *db*/*db* animals (Chow et al., 2007). Immunohistochemistry results from kidney biopsies of patients with T2DM showed overexpression of CCL2 and CCR2 in the glomeruli of these patients compare to healthy kidneys (Tarabra et al., 2009). This was accompanied with reduced nephrin expression in cultured podocytes from T2DM patients (Tarabra et al., 2009). Moreover, in the STZ- model of diabetes, induction of diabetes increased albuminuria in CCL2^+/+^ mice, which was significantly reduced in CCL2-deficient mice. Together these studies suggest a pathogenic role of the CCL2/CCR2 axis in the development of diabetic nephropathy (Tarabra et al., 2009) and targeting this pathway could be a potential therapeutic avenue to reduce common diabetic complications associated with aberrant infiltration of leukocytes.

Other chemokines and their receptors are also changed in T2DM. CX3CL1 was found at higher levels in the subcutaneous adipose tissue of patients with T2DM and in obese people when compared to patients with normal weight and also was shown to modulate monocyte adhesion to adipocytes (Shah et al., 2011). CCL5 (RANTES) was also found at higher levels in the circulation of 236 patients with T2DM, 242 individuals with impaired glucose tolerance (IGT) but not in 244 individuals with normal glycemic control (Herder et al., 2005). However, higher circulating levels of CCL5 were not significantly associated with other inflammatory variables and metabolic parameters, suggesting more studies are required to evaluate the novel hypothesis that CCL5 is a risk factor for T2DM.

Infiltration of inflammatory leukocytes in adipose tissue plays an important role in the development of insulin resistance. The inflammatory cytokines secreted by these cells interfere with insulin signaling and decrease glucose uptake in peripheral tissues (Asghar and Sheikh, 2017). Studies looking at ways to down-regulate aberrant recruitment of leukocytes as a mean to control inflammation and therefore restore insulin sensitivity have targeted chemokine receptors such as CCR5 and CCR2. CCR5 a chemokine receptor expressed by T-cells and macrophages, plays a critical role in recruitment and polarization of macrophage in inflammation. Kitade et al. (2012) showed that a higher gene expression of CCR5 in the white adipose tissue of High Fat Diet (HFD)-induced obese mice was concomitant to an accumulation of macrophages in this tissue. Importantly, mice deficient for CCR5 in their myeloid lineage did not develop insulin resistance and diabetes normally induced by HFD. Loss of CCR5 was associated with a reduction in total adipose tissue macrophage content and polarization of macrophages toward and an anti-inflammatory M2-dominant phenotype in the adipose tissue (Kitade et al., 2012).

The administration of a dual CCR2/CCR5 antagonist has shown to improve obesity-associated insulin resistance and glucose intolerance via reducing macrophages and CD8^+^ T-cell numbers in the white adipose tissue of HFD-fed mice, indicating that blocking both CCR2 and CCR5 has potential to maintain both metabolic and immune homeostasis in obesity-induced inflammation (Huh et al., 2018).

Overall, these studies clearly demonstrate how up-regulation of chemokine pathways, which in turn leads to aberrant recruitment of leukocytes in different tissues is contributing to some of the common diabetic complications and targeting this pathophysiological process could be a promising therapeutic advance. However, further research is required to fully characterize the interactions and redundancy of chemokine with chemokine receptors in T2DM.

## Innate and Adaptive Immune Cell in T2DM

In this section, we will explore how the recruitment of innate and adaptive immune cells changes in obesity and T2DM as summarized in Table 1. Obesity-induced inflammatory events originating from the adipose tissue such as production of local inflammatory molecules are responsible for the activation of immune responses and progression of systemic inflammation in patients with T2DM (Richardson et al., 2013).

**TABLE 1:** A summary of studies discussed in this review assessing obesity/T2DM-related changes to the trafficking of innate and adaptive immune cells.

	Effect of obesity/T2DM on cell recruitment	Potential mechanisms	References
Monocyte/macrophage	↑ recruitment of macrophages in retinas of STZ-induced diabetic rats	CCL2-CCR2 axis	Rangasamy et al., 2014
	↑ recruitment of macrophages in kidney of STZ-induced diabetic mice	CCL2-CCR2 axis	Tarabra et al., 2009
	↑ adhesion of monocyte to adipocytes in the subcutaneous adipose tissue of patients with T2DM and obesity **(Human study)**	↑ CX3CL1 expression in adipose tissue	Shah et al., 2011
	↑ recruitment of macrophages in white adipose tissue of HFD-induced obese mice	↑ CCR5 expression in white adipose tissue	Kitade et al., 2012
	↑ levels of F4/80 and CD11c mRNA expression in adipose tissue of HFD fed mice	↑ Adipose tissue expression of ICAM-1, VCAM-1, CCL2, CXCL14	Kawanishi et al., 2010
	↑ expression of F4/80 in adipose tissue of HFD fed mice	↑ Adipose tissue expression of mRNA for ICAM-1	Brake et al., 2006
	↓ RPMs in db/db mice ↑ Increased M2 polarization	Ongoing chronic inflammation in the peritoneal cavity in db/db mice	Liu et al., 2012
	*In vitro* ↓ adhesion and phagocytosis capacity of RPMs from in *db/db* mice	abnormal microenvironment in db/db mouse	Liu et al., 2012)
	↓ monocyte/macrophages in lung tissue of diabetic DPP4^H/M^ mice following infection by *MERS-CoV*	↓ lung tissue expression of *Ccl2* and *Cxcl10*	Kulcsar et al., 2019
DCs	↑ CD11c^high^F4/80^low^ DCs in mice visceral adipose tissue in HFD-induced obese mice	Not described	Bertola et al., 2012.
	↑ CD11c^+^CD1c^+^ cDCs in obese human subcutaneous adipose tissue **(Human study)**	Not described	Bertola et al., 2012.
	↑ CD11c^+^ cDCs numbers in the adipose tissue of HFD-fed mice	Not described	Chen et al., 2014
Neutrophils	↑ trafficking of neutrophil into mice visceral adipose tissue after 3 days on HFD	↑ CD11b surface expression on neutrophils	Elgazar-Carmon et al., 2008
	↑ recruitment of neutrophils into the adipose tissue of HFD-fed mice after 3 days on HFD (sustained infiltration for up to 90 days on HFD)	↑ expression and activity of neutrophil-secreted elastase in the adipose tissue of HFD-fed mice	Talukdar et al., 2012
CD8^+^ T-cells	↑ infiltrated CD8^+^ effector T-cells in visceral adipose tissue in HFD-fed mice	Activation of CD8^+^T cells by endogenous stimuli localized in the adipose tissue	Nishimura et al., 2009.
	↓ lower numbers of CD8^+^ T-cells in their brains in *db/db* mice following infection with *West Nile virus*	↓ expression of E-selectin and ICAM-1 in *db/db* brains	Kumar et al., 2014
CD4^+^ T-cells	↓ CD4 + T-cells in lung tissue of diabetic DPP4^H/M^ mice following infection by *MERS-CoV*	↓ lung tissue expression of *Ccl2* and *Cxcl10*	Kulcsar et al., 2019
Tregs	↓ regulatory T-cells in visceral adipose tissue of HFD-fed mice	Not described	Nishimura et al., 2009.
	↓ CD4^+^ Tregs HFD-fed mice	↓ levels of adiponectin in obese fat	Feuerer et al., 2009; Ilan et al., 2010.
B-cells	↑ number of B-cells in the bone marrow of HFD-fed mice	Not described	Trottier et al., 2012
	↓ number of B-cells in the bone marrow of HFD-fed mice	lower expression of Pax5 in the bone marrow of HFD-fed mice	Chan et al., 2012
Granulocytes	↓ granulocytes in the alveolar airspace of stz-induced diabetic mice following infection by *Klebsiella pneumoniae*	↓ levels of (CXCL1, CXCL2) and (IL-1β, TNFα) in lung tissue	Martinez et al., 2016

### Innate Immune Cells

#### Monocytes and Macrophages

Innate immune cells play a critical role in the early stage of adipose tissue inflammation in T2DM (Weisberg et al., 2003). Monocytes derived from the bone marrow remain in the circulatory system for 1–2 days before they migrate into peripheral tissues, where they turn into fully mature resident macrophages (Martinez-Pomares et al., 2003) either with a M1 pro-inflammatory or M2 anti-inflammatory phenotype in response to local factors (Mosser, 2003). In a recent *in vivo* study, an acute decrease in number of circulating monocytes was synchronized with a concomitant infiltration of macrophages into visceral adipose tissue, therefore demonstrating dynamic changes in blood monocyte trafficking at the early stages of HFD-induced diabetes (Liu et al., 2020). Higher levels of F4/80 and CD11c mRNA expression in adipose tissue of HFD fed mice, representative of a M1 polarization, were attributed to an elevated gene expression of ICAM-1, VCAM-1, CCL2 and CXCL14 in this tissue, which facilitates macrophage trafficking (Kawanishi et al., 2010). Similarly, Brake et al., found higher levels of mRNA for ICAM-1 in mice on a 3-week HFD, as well as an increased expression of F4/80 in their adipose tissue (Brake et al., 2006). The activation of M1 macrophages is associated with release of pro-inflammatory mediators, such as TNFα and IL-6 (Biswas and Mantovani, 2010). Using an HFD-induced rat model of T2DM, Shanaki et al. reported increased serum and adipose tissue levels of IL-6 and TNFα in T2DM rats compared to control animals. This pro-inflammatory shift was linked to higher fasting plasma glucose levels and insulin resistance in the diabetic animals (Shanaki et al., 2020). The impact of long-term diabetes on the functions of macrophages was investigated using a 5-month old *db/db* mice model of T2DM (Liu et al., 2012). In this study, the authors focused on resident peritoneal macrophages (RPMs) to investigate potential changes of their function, phenotype and migratory capacity. RPMs numbers were reduced in *db/db* mice compared to C57BL/6 control mice and most were preferentially polarized toward a M2 phenotype. Since M2 macrophages exhibit immunosuppressive functions and therefore contribute to the resolution of harmful inflammation, their increased proportion in the *db/db* mice may be caused by an effort to control the chronic low-grade inflammation observed in the peritoneal cavity of these mice (Liu et al., 2012). Furthermore, in this study, *in vitro* assessment of *db/db* RPMs highlighted their decreased adhesion capacity along with a decreased phagocytosis ability compared to wild-type RPMs. These observations validate that diabetes and obesity are associated with immune dysfunction which predispose these patients to an increased susceptibility to infections (Liu et al., 2012).

Human studies also indicate a role of macrophages in mediating insulin resistance. Indeed, higher numbers of circulating leukocytes are found in patients with T2DM along with higher Free Fatty Acid (FFA) and IL-6 circulating levels (van Beek et al., 2014). Elevated number of total leukocytes counts in patients with T2DM was also characterized in other studies (Kizilgul et al., 2018; Palella et al., 2020) and were often indicative of macro- and micro-vascular complications and diabetes duration (Papazafiropoulou et al., 2010; Moradi et al., 2012). In particular, Wouters et al. demonstrated higher numbers of circulating classical monocytes and this was associated with higher presence of M1 macrophages in the white adipose tissue of individuals with obesity compared to individuals with normal weight (Wouters et al., 2017). In addition, impaired glucose tolerance was associated with the presence of crown-like structures (marker of adipose inflammation) in the adipose tissue of patients with obesity and T2DM compare to patients with obesity and normal glucose tolerance (van Beek et al., 2014).

Most of the current observations seem to indicate a crucial role of macrophage infiltration in the adipose tissue of patients with obesity and T2DM. However, it is not entirely clear what mechanisms mediate recruitment of macrophages in these tissues and more research is needed to understand whether macrophage infiltration could be limited to improve insulin sensitivity and glucose tolerance and therefore prevent diabetic complications.

#### Neutrophils

Neutrophils are the most abundant subsets of leukocytes of the innate immune system in humans and are the first to infiltrate inflamed tissue and promote subsequent recruitment of other leukocytes such as monocytes (Soehnlein et al., 2008). The number of circulating neutrophils in patients with T2DM increases in comparison to age- and gender-matched healthy controls (Huang et al., 2019). However, neutrophils isolated from patients with T2DM displayed normal migratory capacity and phagocytic rate (Huang et al., 2019). Neutrophil trafficking into murine visceral adipose tissue was reported after 3 days on HFD. The same study reported an absence of neutrophils in the adipose tissue after 7 days on HFD and that neutrophil adhesion to mouse adipocytes depends on their activation state (Elgazar-Carmon et al., 2008). Here, the degree of CD11b surface expression on neutrophils correlated with their capacity to adhere. However, the mechanisms responsible for the absence of neutrophils in the adipose tissue after 7 days on HFD was not explained in this study (Elgazar-Carmon et al., 2008). Similarly, Talukdar et al. (2012) found an early recruitment of neutrophils into the adipose tissue of HFD-fed mice after 3 days. Here, the time course of neutrophil infiltration into the adipose tissue showed a sustained infiltration in the adipose tissue for up to 90 days on HFD (Talukdar et al., 2012). According to this study, the trafficking of neutrophils was associated with an elevated expression and activity of neutrophil-secreted elastase in the adipose tissue of HFD-fed mice which increased after only 3 days of HFD and remained high after 12 weeks. Elastase is a neutrophil-specific protease which can promote inflammatory responses (Pham, 2006) and inhibition of neutrophil elastase in HFD-induced obese mice improved glucose tolerance and reduced trafficking of neutrophils into the adipose tissue (Talukdar et al., 2012).

#### Dendritic Cells

Dendritic cells (DCs) are antigen-presenting immune cells that mediate lymphocytes polarization into effector cells (Yu and Martin-Gayo, 2019). In humans, two major subtypes of DCs are identified according to their markers expression: conventional or myeloid DCs (cDCs) (CD11c^+^ CD1c^+^ CD141^+^) and plasmacytoid DCs (pDCs) (CD11c^–^ CD123^+^) (Tamura et al., 2005; Gilliet et al., 2008; Sundara Rajan and Longhi, 2016). In mice, two major DC subsets have also been described: CD11c^*low*^B220^+^ (pDCs) and CD11c^high^B220^–^ cells that include cDCs (O’Keeffe et al., 2002). The number of circulating cDCs increases in obese post-menopausal women with T2DM compared to age-matched healthy women and a smaller increase was observed for pDCs (Musilli et al., 2011). This change in DCs numbers in the circulation suggests that DCs might contribute to pathological vascular remodeling (Musilli et al., 2011). Mráz et al. reported a decrease in the number of total DCs in the subcutaneous adipose tissue from patients with T2DM compared to non-diabetic individuals. In contrast, the number of pDCs was increased in the subcutaneous adipose tissue of the T2DM group. These differences suggest a potential role of pDCs in the development of T2DM-associated adipose tissue low-grade inflammation (Mráz et al., 2019). Bertola et al. investigated the role of DCs in the regulation of adipose tissue inflammation in a murine HFD-induced obesity model and in two cohorts of obese subjects (Bertola et al., 2012). The authors in this study only found CD11c^high^B220^–^ DCs in the visceral adipose tissue of lean mice. In contrast, obesity was associated with the presence of CD11c^high^F4/80^low^ DCs in murine visceral adipose tissue, and CD11c^+^CD1c^+^ cDCs in human subcutaneous adipose tissue (Bertola et al., 2012). CD11c^high^B220^–^ DCs resident in the adipose tissue of lean mice participate in the differentiation of naive CD4^+^ T-cells into effector T-cells with a predominance of Th1 cells over Th17 cells. In contrast, the adipose tissue of HFD-induced insulin resistant mice displayed a switch from Th1 towards a Th17 phenotype (Bertola et al., 2012). In addition, the presence of CD11c^+^CD1c^+^ cDCs in the subcutaneous adipose tissue of obese subjects correlated with CD1c expression and a concomitant skew towards a Th17 T-cell phenotype. Finally, CD1c expression strongly correlated with insulin resistance in patients with a high Body Mass Index (BMI) (Bertola et al., 2012). Altogether, these observations suggests an important role of inflammatory DCs in obese and diabetic adipose tissue inflammation by switching T-cell responses toward Th17 responses, which is associated with insulin resistance (Bertola et al., 2012). These observations were confirmed by another group who showed that CD11c^+^ cDCs infiltrated the adipose tissue of HFD-fed mice and also secrete high levels of IL-6 and IL-23, which promoted a Th17 T-cell phenotype (Chen et al., 2014). The presence of DCs in the visceral adipose tissue of HFD-mice can also induce the formation of crown-like structures which enclose macrophages and were linked to adipose tissue inflammation and insulin resistance (Stefanovic-Racic et al., 2012). Depletion of DCs results in loss of adipose tissue macrophage infiltration, and this could be restored by DC replacement in DC-null mice (Stefanovic-Racic et al., 2012). All together, these studies show the importance of DCs in determining the immune phenotype of lean versus obese or diabetic adipose tissue.

### Adaptive Immune Cells

The mucosal immune system is a compartment of the adaptive immune system which is located near the surface, where most pathogens invade, providing the first line of defense (Janeway et al., 2001). The gut microbiota is key to the development and modulation of mucosal immune responses and maintains perfect balance between commensal flora and pathogens, as well as the microbiota and the immune system (Wang and Li, 2015). The alteration of such balance is called dysbiosis (Pagliari et al., 2018). Given that the pancreas does not have its own microbial collection, the gut microbiota may be involved in the pathogenesis of pancreatic disorders such as pancreatitis (Signoretti et al., 2017). Recently, Guo et al. (2017) demonstrated that HFD was able to alter gut microbial communities and increase circulating pro-inflammatory cytokines, such as TNFα, IL-6 and IL-1β. In addition, recent evidence demonstrated that intestinal dysbiosis may also cause alterations in the Th17 cells/Tregs balance which are responsible for the development inflammatory disease including obesity-related T2DM (Luo et al., 2017).Thus, understanding the mechanisms responsible for this alteration will allow to develop novel translational therapeutic targets to potentially treat these inflammatory diseases (Pagliari et al., 2018). In this section, we mostly focus on T- and B-cells as very little is known on mucosa-associated homing mechanisms in the context of T2DM.

#### T-Cells

Although, earlier studies focused on the role of innate immunity (macrophages) as the major cause of chronic low-grade inflammation in T2DM, the adaptive immune system also plays a role in progression of T2DM (Zhou et al., 2018). Evidence shows that B-cells are the first to infiltrate the adipose tissue of mice on HFD, quickly followed by T-cells and finally an accumulation of macrophages leading to insulin resistance, but no changes in resident macrophages populations (Duffaut et al., 2009). Nishimura et al. (2009) found larger number of infiltrated CD8^+^ effector T-cells along with reduced numbers of CD4^+^ helper and regulatory T-cells in visceral adipose tissue of HFD fed mice). In these conditions, a majority of CD8^+^ T-cells infiltrate the adipose tissue thereby promoting recruitment and activation of macrophages. This sequential accumulation of leukocytes is clearly linked with glucose intolerance and a decrease in insulin sensitivity in wild-type animals (Duffaut et al., 2009). Studies in the lymphocyte-deficient *RAG2^–/–^* knockout mouse provide strong evidence for the role of lymphocytes in HFD-mediated adipose tissue inflammation. Unexpectedly, lymphocyte-deficient animals displayed striking accumulation of macrophages and NK cells in the adipose tissue compared to wild-type mice (Duffaut et al., 2009). This accumulation of NK cells highlights an overreaction of the innate immunity in absence of the adaptive immune system. Indeed, NK cells have potent cytotoxic effector functions and produce chemokines and cytokines that can recruit macrophages (Duffaut et al., 2009). The exaggerated recruitment of macrophages in lymphocyte-deficient mice demonstrates that early lymphocyte infiltration could be considered a protective process to decrease adipose tissue inflammation and suggests adipose tissue as a site of dynamic innate and adaptive immune system during diet-induced obesity and insulin resistance (Duffaut et al., 2009).

According to recent reports, the changes in quantity and polarization of adipose tissue T-cells during weight gain is a key regulator of systemic insulin sensitivity (Deng et al., 2017). HFD is associated with increased numbers of Th1 CD4^+^ T-cells and decreased numbers of CD4^+^ Tregs in the adipose tissue (Feuerer et al., 2009; Ilan et al., 2010). The depletion of Tregs using diphtheria toxin in mice leads to an induction of genes encoding inflammatory cytokines such as TNFα and IL-6 in visceral adipose tissue and enhance levels of fasting insulin (Feuerer et al., 2009). On the other hand, in the same study, increasing the quantity Tregs in the adipose tissue using a Tregs–enriched HFD-fed mice model, improved insulin resistance and glucose tolerance (Feuerer et al., 2009). Since, the gene expression of IL-10 was increased in the adipose tissue of Tregs–enriched animals, the metabolic changes observed can be attributed to IL-10 synthesis by Tregs in the adipose tissue of these mice (Feuerer et al., 2009). These findings suggest a strong therapeutic potential for Tregs to suppress inflammation and improve insulin action in obesity and T2DM. It is therefore key to understand how Tregs migratory capacity and infiltration in the adipose tissue could be manipulated in patients. In addition, further studies are required to fully characterize the involvement of different T-cell subsets and characterize the intricate balance between innate and adaptive immune regulations.

#### B-Cells

B-cells have been shown to play important roles in many chronic inflammatory and autoimmune conditions (Yanaba et al., 2008; Mariño and Grey, 2012) but only recently has their role been revealed in obesity-associated insulin resistance. B-cells infiltrate the white adipose tissue in diet-induced obesity models and contribute to insulin resistance (Winer et al., 2011). The data on the development of B-cells in the bone marrow of obese models is controversial. Indeed, B-cells numbers in the bone marrow of mice on HFD were significantly increased after 90 days (Trottier et al., 2012). However, number of B-cells in the bone marrow were reduced in C57BL/6 mice on HFD for 210 days in another study (Chan et al., 2012). This reduction was associated to a lower expression of Pax5 in the bone marrow of HFD-fed mice (Chan et al., 2012). Differences between these studies may be due the type and duration of HFD. B-cell infiltration in the white adipose tissue of C57BL/6 mice peaks at around 3–4 weeks of HFD (Duffaut et al., 2009). The importance of B-cells in adipose tissue inflammation has been studied *in vivo* using B-cell null mice (μMT mice). Attenuated inflammation in visceral adipose tissue of μMT mice was shown by a reduced infiltration of macrophages. In this study, following 15 weeks of HFD, serum glucose levels were unchanged in obese μMT mice while fasting glucose levels increased in the control wild-type group (DeFuria et al., 2013). These data support the conclusion that B-cells regulate macrophage infiltration into the adipose tissue during inflammation and this contributes to insulin resistance.

## Immune Response Following Inflammatory Stimulus in T2DM

Hyperglycemia in diabetes can impair immune response to pathogens such as, fungi, bacterial and viral infections (Hostetter, 1990; Javid et al., 2016; Kulcsar et al., 2019). As a result, patients with diabetes are more susceptible to infections (Berbudi et al., 2020). Although the plasma concentration of sCAMs tends to be generally higher in patients with T2DM compared to healthy controls, their expression is differently affected in response to inflammatory pathogens in patients with T2DM. Indeed, plasma levels of sE-selectin, sVCAM-1 and sICAM-1 after intravenous injection of *E.coli* lipopolysaccharide (LPS) were lower in patients with T2DM compared to healthy volunteers. This study revealed that patients responded with an attenuated up-regulation of sCAMs even though the basal plasma concentration of these adhesion molecules were generally higher in diabetic individuals compared to healthy controls (Andreasen et al., 2010). Also, in this study, T2DM was associated with less pronounced LPS-induced cytokine responses. This weaker cytokine response following inflammatory stimuli in T2DM is also shown *in vitro* with cultures of peripheral blood mononuclear cells (PBMCs). Stimulation of PBMC from patients with diabetes with LPS and *Burkholderia pseudomallei* lead to a lower production of IL-1β and IFNγ respectively, compared to PBMCs from healthy donors (Mooradian et al., 1991; Tan et al., 2012). All together these findings may explain the immune dysfunction and increased risk of infections associated with T2DM (Andreasen et al., 2010). However, the evidence demonstrating changes in leukocyte recruitment following pathogen invasion in T2DM is rare and mostly limited to animal experimental studies. Accordingly, *db/db* mice infected with West Nile virus, had lower numbers of CD45^+^ leukocytes and CD8^+^ T-cells in their brains compared to wild-type mice (Kumar et al., 2014). Effector CD8^+^ T-cells are necessary to limit viral load and therefore a lack of CD8^+^ T-cells infiltration, which may be caused by a decreased migratory capacity or changes in the brain vasculature, lead to an increased viral burden in the brain of infected mice (Shrestha and Diamond, 2004; Kumar et al., 2014). This defect in leukocyte recruitment was in fact due to a reduced expression of E-selectin and ICAM-1 in *db/db* brains which failed to properly support the CD8^+^ T-cells adhesion and migration (Kumar et al., 2014). Furthermore, impaired recruitment of leukocytes is not only due to changes on leukocytes themselves and in the local environment but can also be attributed to attenuated cytokine and chemokine production in diabetic mice. In STZ-diabetic mice infected by *Klebsiella pneumoniae*, which causes pneumonia (Bengoechea and Sa Pessoa, 2019) lower numbers of granulocytes were found in the alveolar airspace along with attenuated chemokines (CXCL1, CXCL2) and cytokines (IL-1β, TNFα) levels in lung tissue when compared to control mice (Martinez et al., 2016). Moreover, a recent study investigated the impact of T2DM on respiratory infection caused by Middle East respiratory syndrome coronavirus (MERS-CoV) (Kulcsar et al., 2019). In this study, humanized DDP4 mice were susceptible to MERS-CoV and T2DM was induced by HFD (Kulcsar et al., 2019). Following infection with MERS-CoV, diabetic *DPP4*^*H/M*^ mice displayed weight loss and had a longer phase of severe disease with delayed recovery. Importantly, lung tissue analysis in the diabetic mice showed a decreased number of macrophages, CD4^+^ T-cells, and lower expression of TNFα and IL-6 in the HFD group, compared to control *DPP4*^*H/M*^ mice following infection (Kulcsar et al., 2019). These results suggest that MERS-CoV infection in patients with T2DM diabetes may develop more severe disease as a result of a dysregulated immune response targeting not only migratory capacities of leukocytes but the local environment by modulating secreted factors and expression of adhesion molecules. Maximal endothelial cell adhesion molecule expression or chemokine/cytokine receptor downregulation/internalization in chronic inflammatory conditions could theoretically cause an impaired immune response to an infection. In addition to alternative mechanisms, it is also plausible that insulin could have agonist and antagonist effects on the same signaling pathway based on its concentration and receptor expression state (i.e., the “U-shaped” biologic dose-response curve) (Calabrese and Baldwin, 2001). In general, it seems that high glucose levels are associated with impaired expression of adhesion molecules, cytokines and the chemokines supporting efficient leukocyte migration during an inflammatory response to infections in T2DM conditions. This is in contrast with studies mentioned above which show how basal expression of adhesion molecules and chemokines/chemokine receptors is increased in different vascular beds and leukocytes in T2DM conditions. The defect in cytokine response to pathogen in T2DM is partially related to insulin deficiency and recently has been discussed by Tessaro and Martins (Tessaro et al., 2017). In their study, insulin increased TNFα and IL-6 release by bone marrow-derived macrophages from diabetic C57BL/6 mice following LPS stimulation. This finding supports the idea that insulin is crucial to induce a proper immune reaction in response to inflammatory stimuli. However, in the same study insulin inhibited LPS-induced pro-inflammatory cytokine secretion by peritoneal macrophages from diabetic mice (Tessaro et al., 2017). Together findings suggest that beyond its glucose modulatory effects, insulin also has distinctly immunomodulatory effects in macrophages and these urgently require to be fully understood.

## Therapeutic Targeting of Leukocyte Trafficking in T2DM

Given that T2DM is a chronic inflammatory disorder, characterizing and targeting possible inflammatory pathways could be efficient to prevent and control diabetes and its vascular complications.

### Chemokine and Cytokine Inhibition

Chemokine and cytokine pathways are known to govern the trafficking of leukocytes into peripheral tissues (Yao et al., 2014). Potential antagonists targeting chemokine receptors and drugs blocking inflammatory cytokines have been developed and tested in many inflammatory conditions, but little is known about their efficacy in obesity or T2DM and associated complications. In this section, we will discuss some of these targets.

CC-chemokine ligand 2 and CCL5 are key mediators of monocyte recruitment induced by high glucose levels via their receptor CCR2 and CCR5 (Nunemaker et al., 2014). Oral administration of RO5234444, a CCR2 antagonist, to *db/db* mice, reduced infiltration of monocytes in the glomerulus, resulted in preservation of glomeruli podocytes numbers and reduced albuminuria (Sayyed et al., 2011). Similarly, intraperitoneal administration of TAK-779, a dual inhibitor of chemokine receptors CCR2 and CCR5, reduced macrophage infiltration and expression of ICAM-1 in the retinas of STZ-diabetic mice (Monickaraj et al., 2020). These studies clearly indicate that blocking key recruitment chemokine receptors such as CCR2 and/or CCR5 is a promising therapeutic avenue to ameliorate common diabetic complications such as nephropathy and retinopathy. Those compounds are currently pursued in human clinical trials as well as others such as INCB8761/PF-413630, which are a new series of CCR2 antagonists that are orally bioavailable (Xue et al., 2011).

Due to their pro-inflammatory nature, IL-1β and TNFα actions have been widely studied in many inflammatory conditions and their blockade resulted in improvements in T2DM-related conditions such as chronic kidney disease (Lei et al., 2019) and pancreatic islet inflammation (Zha et al., 2016). However, it remains unsure whether targeting these cytokines affects leukocyte infiltration in peripheral tissues in T2DM. Circulating levels of IL-1β are higher in T2DM (Reinehr et al., 2016) and macrophages are the primary source of IL-1β in obesity-induced inflammation (Gao et al., 2014). Administration of LY2189102, a neutralizing IL-1β antibody, in patients with T2DM, improved glycemic control and demonstrated significant anti-inflammatory effects by lowering circulating IL-6 levels when compared with placebo treatment (Sloan-Lancaster et al., 2013). Moreover, islets from IL-1β-deficient mice exposed to high glucose *in vitro*, produced lower IL-6 and chemokines compared to wild-type islets (Ehses et al., 2009). Thus, efficacy of IL-1β blocking on inflammatory biomarkers and glycemic control raises the possibility of its use as a treatment in T2DM and other inflammatory conditions.

Although, anti-TNFα is an approved medication for some patients with rheumatoid arthritis (Katsumata et al., 2019), the evidence showing the efficacy of this drug in T2DM is conflicting. Indeed, anti-TNFα treatment using recombinant soluble TNFα receptor-immuno-globulin G increased insulin sensitivity in obese rodents (Hotamisligil et al., 1993). However, treatment with Ro 45-2081, a TNFα antagonist (recombinant fusion protein that consists of the soluble TNF-receptor linked to the Fc portion of human IgG1), had no effect on blood glucose levels and insulin-mediated glucose uptake in patients with T2DM (Paquot et al., 2000). The inefficiency of Ro 45-2081 to control blood glucose in patients with T2DM suggest that in addition its endocrine action, TNFα may also act through an autocrine or paracrine route. Consequently, sole neutralization of circulating TNFα might not be enough to observe improvements on insulin action (Paquot et al., 2000).

### Anti-integrins

The importance of integrins in leukocyte adhesion and arresting clearly offer potential therapeutical avenues in relapsing inflammatory conditions such as rheumatoid arthritis (von Andrian and Engelhardt, 2003) and multiple sclerosis (Chaudhuri and Behan, 2003). Neutralization of integrins has been tested in murine experimental models of diabetes, particularly for the treatment and/or prevention of diabetic complications (Barouch et al., 2000; Iliaki et al., 2009; Miyachi et al., 2017). However, data showing efficacy in human clinical trials is not available in the context of T2DM.

The expression of CD11a, CD11b, and CD18 integrins is increased on the surface of neutrophils from STZ-induced diabetic rats (Barouch et al., 2000). This increase was associated with an enhanced adhesion of diabetic neutrophils to rat EC monolayers *in vitro.* Pre-treatment of leukocytes with either anti-CD11b or anti-CD18 antibodies significantly lowered the proportion of adherent diabetic neutrophils (Barouch et al., 2000). In the same study, systemic administration of anti-CD18 F(ab′)_2_ fragments to STZ-induced diabetic rats significantly decreased diabetic retinal leukostasis (Barouch et al., 2000). Furthermore, intraperitoneal administration of anti-α_4_ integrin neutralizing antibody to a STZ-induced rat model of diabetes retinopathy attenuated leukocyte adhesion to the retina and suppressed TNFα expression and NF-κB activity in the retina of treated rats (Iliaki et al., 2009). Notably, blockage of VLA-4 using a neutralizing antibody in HFD-fed mice attenuated myeloid cell accumulation in the liver and improved systemic glucose tolerance in treated mice compared to control HFD-fed mice (Miyachi et al., 2017). Overall, all these animal data identify the integrins as functional adhesive molecule in diabetic complications and provides a potential target for the prevention and/or treatment of the disease.

A humanized anti–VLA-4 monoclonal antibody (Natalizumab) was clinically beneficial in the treatment of Crohn’s disease (Lew and Stoffel, 2003), and rheumatoid arthritis (von Andrian and Engelhardt, 2003). However, VLA-4 also has key regulatory roles in immune response, including the formation of the immune synapse (Mittelbrunn et al., 2004) and the differentiation of Th1 T-cells (Isobe et al., 1997). Obviously, the long-term administration of anti-integrins drugs to patients with inflammatory conditions may have undesirable or unexpected effects and more studies are required to fully understand their action.

### Fatty Acids

Long-chain *n*–3 polyunsaturated fatty acid (*n*–3 PUFA) such as eicosatetraenoic acid (EPA) and docosahexaenoic acid (DHA), found in marine fish oils, are able to down-regulate the activity of NF-kB directly and therefore reduce the production of inflammatory cytokines (Xu et al., 2001). *n*–3 PUFA can also control leukocyte recruitment during inflammation and display immunomodulatory properties (Yates et al., 2011). Inclusion of *n*–3 PUFA in HFD prevented macrophage infiltration into the adipose tissue of *db/db* mice (Todoric et al., 2006). Interestingly, *n*–3 PUFA efficiently prevented the HFD-induced downregulation of adiponectin circulating levels normally observed in *db/db* (Todoric et al., 2006). These data therefore suggest that the beneficial effects of *n*–3 PUFA on diabetes could be mediated by their effect on adipose tissue inflammation, which could in turn contribute to improving insulin sensitivity (Oliver et al., 2010). However, much of the human evidence examining the beneficial effects of n–3 PUFA in patients with T2DM have limited follow-up periods (Vessby et al., 2001). As a result, it is unclear whether *n*–3 PUFA supplementation has long lasting beneficial effects and the exact mechanisms of *n*–3 PUFA action and how they modulate the adipose tissue, macrophages and even T-cells remain unknown.

### Insulin

Several studies suggest a direct anti-inflammatory action of insulin irrespectively of its glucose modulating capacity as mentioned here in previous sections (Hyun et al., 2011). Insulin was found to attenuates the activity of NF-κB and MCP-1 on human aortic ECs *in vitro* (Aljada et al., 2001). Insulin infusion in individuals with obesity reduced plasma sICAM-1 and CCL2 levels (Dandona et al., 2001). In addition, treatment of human monocytes with insulin *in vitro* promoted the secretion of IL-8 (CXCL8), a potent chemoattractant for neutrophils (Dandona et al., 2001). All together, these findings suggest a possible regulatory effect of insulin on leukocyte trafficking via regulating chemokine and cytokine secretion as well as modulating adhesion molecules shedding. However, more studies are required to clarify the effect of insulin on leukocyte migration by including *in vitro* models taking into account the high glucose levels observed in some patients with T2DM, their associated complications and BMI to accurately determine the mechanism of insulin action and lift the controversy in this field.

### Adiponectin and PEPITEM

Since obesity is a key feature in T2DM, studies demonstrate that changes in adipose tissue induce dysregulation in adipokines such as adiponectin and receptors involved in lipid metabolism such as Proliferator–activated receptor γ (PPAR-γ) (Bermudez et al., 2010). Adiponectin is well characterized as an insulin-sensitizing hormone as well as an anti-inflammatory adipokine (Ruan and Dong, 2016). Circulating levels of adiponectin are decreased in patients with obesity and T2DM (Hotta et al., 2000; Engeli et al., 2003), suggesting that dysregulation of adiponectin may be relevant to obesity-linked endothelial dysfunction in these individuals (Cho et al., 2002; Katsiki et al., 2017). In addition, adiponectin is also a regulator of adhesion molecules on ECs (Ouchi et al., 1999). Treating ECs with adiponectin inhibited the expression of adhesion molecules such as VCAM-1, E-selectin, and ICAM-1 *in vitro* (Ouchi et al., 1999) and *in vivo* (Ouedraogo et al., 2007). We also know that adiponectin is able to regulate T-cell trafficking during inflammation in a novel pathway characterized in our laboratory a few years ago (Chimen et al., 2015). In this novel pathway, adiponectin induces the release of the novel PEPtide Inhibitor of Trans-endothelial Migration (PEPITEM) by B-cells via signaling through the adiponectin receptors (AdipoR1/2). PEPITEM induces the release of sphingosine-1-phosphate (S1P) by ECs via binding with endothelial cadherin-15 (CDH15). In turn, S1P inhibits T-cell transmigration by preventing integrin activation (Chimen et al., 2015). The PEPITEM/Adiponectin pathway is dysregulated in inflammatory conditions such as Type 1 Diabetes Melitus, Rheumatoid Arthritis and in older adults. Dysregulation of this pathway is caused by a lack of AdipoR1/2 expression on B-cells in these patient groups leading to insufficient secretion of PEPITEM and consequently allowing aberrant T-cell trafficking. PEPITEM shows great therapeutic potential since it is an endogenous peptide and developing strategies to restore this pathway could help regain control on chronic inflammation. However, more work is needed to understand the full profile of T-cell subsets recruitment in adipose and pancreatic tissues in T2DM as this would allow to characterize whether PEPITEM could be used as a therapeutic avenue in T2DM.

### PPAR Agonists

Proliferator–activated receptor-γ is a member of the PPAR family of nuclear receptors (Braissant et al., 1996) and its activation is associated with the induction of glucoregulatory molecules and enhanced insulin sensitivity (Olefsky, 2000). Pioglitazone is a PPARγ agonist that enhances the action of insulin mainly by promoting glucose utilization in peripheral tissues (Yki-Järvinen, 2004). Treatment of patients with T2DM with pioglitazone rapidly reduced systemic inflammation in those patients who were also receiving angiotensin II receptor blockers. This trial also showed decreased CRP levels accompanied by a reduction in sICAM-1 and sVCAM. These observations uncovered anti-atherogenic effects of the PPAR-γ agonist which may also contribute to a reduction of cardiovascular events in patients at risk such as those with T2DM (Takase et al., 2007).

Current drugs such as IL-1β blockers and thiazolidinediones targeting the metabolic side of T2DM and aiming to restore glucose tolerance have shown to also have anti-inflammatory effects (Yki-Järvinen, 2004; Sloan-Lancaster et al., 2013). However, the evidence showing a potential effect of these drugs on leukocyte recruitment as a way to control inflammation are limited and this area needs further investigations. Blocking cytokines such as TNF- α may reduce inflammation but also renders the host susceptible to infection by silencing the danger signals, which are necessary for adequate immune cell activation (Rider et al., 2016) and maybe even to cancer (reviewed in Dinarello, 2005). On the other hand, anti-cytokine therapy has little or no organ toxicity or gastrointestinal disturbances and so is well tolerated. Therefore, new site-restricted biologics which block inflammatory cytokines only at sites of inflammation are needed (Rider et al., 2016). In addition, it seems that targeting inflammation in T2DM and obesity is promising but still only show partial reduction of disease. This could be explained by the fact that diabetes and obesity-mediated inflammation involve multiple mechanisms and are not necessarily always linked to hyperglycemia. This clearly highlights the need to identify how anti-inflammatory treatments modulate glucose tolerance and the complications associated with T2DM, but also an urgent necessity to understand how inflammation is dysregulated in T2DM and in obesity. More work is clearly required to fully characterize the mechanisms behind the changes in leukocyte phenotype and in local environments which directly influence leukocyte migration. Finally, more specialistic studies from leukocyte trafficking groups are needed to entirely determine the changes in leukocyte migratory capacity and behaviors *in vitro* and *in vivo* to complete the findings showing changes in expression profiles such as those of adhesion molecules.

## Author Contributions

LP wrote the first draft of the manuscript. MC and AT contributed to the manuscript revision, read and approved the submitted version. All authors contributed to the article and approved the submitted version.

## Conflict of Interest

The authors declare that the research was conducted in the absence of any commercial or financial relationships that could be construed as a potential conflict of interest.
